# The Metabolic Core and Catalytic Switches Are Fundamental Elements in the Self-Regulation of the Systemic Metabolic Structure of Cells

**DOI:** 10.1371/journal.pone.0027224

**Published:** 2011-11-18

**Authors:** Ildefonso M. De la Fuente, Jesus M. Cortes, Martin B. Perez-Pinilla, Vicente Ruiz-Rodriguez, Juan Veguillas

**Affiliations:** 1 Instituto de Parasitologia y Biomedicina Lopez-Neyra, CSIC, Granada, Spain; 2 Instituto de Investigación Sanitaria Biocruces, Barakaldo, Bizkaia, Spain; 3 DECSAI: Departamento de Ciencias de la Computacion e Inteligencia Artificial, Universidad de Granada, Granada, Spain; 4 CITIC: Centro de Investigación en Tecnologías de La Información y de las Comunicaciones, Universidad de Granada, Granada, Spain; 5 Departmento de Matemáticas, Facultad de Ciencia y Tecnología, Universidad del País Vasco, Bizkaia, Spain; 6 Departamento de Química-Física, Facultad de Ciencias y Tecnología, Universidad del País Vasco, Bizkaia, Spain; Université Joseph Fourier, France

## Abstract

**Background:**

Experimental observations and numerical studies with dissipative metabolic networks have shown that cellular enzymatic activity self-organizes spontaneously leading to the emergence of a metabolic core formed by a set of enzymatic reactions which are always active under all environmental conditions, while the rest of catalytic processes are only intermittently active. The reactions of the metabolic core are essential for biomass formation and to assure optimal metabolic performance. The on-off catalytic reactions and the metabolic core are essential elements of a Systemic Metabolic Structure which seems to be a key feature common to all cellular organisms.

**Methodology/Principal Findings:**

In order to investigate the functional importance of the metabolic core we have studied different catalytic patterns of a dissipative metabolic network under different external conditions. The emerging biochemical data have been analysed using information-based dynamic tools, such as Pearson's correlation and Transfer Entropy (which measures effective functionality). Our results show that a functional structure of effective connectivity emerges which is dynamical and characterized by significant variations of bio-molecular information flows.

**Conclusions/Significance:**

We have quantified essential aspects of the metabolic core functionality. The always active enzymatic reactions form a hub –with a high degree of effective connectivity- exhibiting a wide range of functional information values being able to act either as a source or as a sink of bio-molecular causal interactions. Likewise, we have found that the metabolic core is an essential part of an emergent functional structure characterized by catalytic modules and metabolic switches which allow critical transitions in enzymatic activity. Both, the metabolic core and the catalytic switches in which also intermittently-active enzymes are involved seem to be fundamental elements in the self-regulation of the Systemic Metabolic Structure.

## Introduction

Living cells are essentially highly evolved dynamic metabolic structures, in which the most complex molecules of Nature are synthesized and destroyed by means of sophisticated self-regulating catalytic cycles.

Cells exhibit a rich variety of reactive dynamic phenomena [1] and millions of biochemical interactions forming one of the most complex self-organized networks [2].

The cellular biochemical reactor presents a surprising molecular crowding in which enzymes are the most outstanding molecules; they are responsible for almost all catalytic transformations, which globally considered constitute cellular metabolism.

One of the most important goals of the post-genomic era is to understand the elementary principles and quantitative laws governing the functional metabolic architecture of the cell.

Intensive studies of protein-protein interactions have shown that in the cellular internal medium, enzymes do not work isolately but forming supramolecular complexes [3] e.g., the analyses of the proteome of *Saccharomyces cerevisiae* have shown that at least 83% of all proteins form complexes -containing from two to eighty-three proteins- and their overall enzymatic structure is formed by a modular network of biochemical interactions between enzymatic complexes [4]. These associations occur in all kinds of cells, both eukaryotes and prokaryotes [5]–[7].

Some associations of various enzymes in large complexes may allow the direct transfer of their common intermediate metabolites from the active site of one enzyme to the catalytic centre of the following enzyme without prior dissociation into the bulk solvent (substrate channelling). This process of non-covalent direct transfer of metabolic intermediates allows for a decrease in the transit time of reaction substrates, originating a faster catalysis through the pathway, preventing the loss of reaction intermediates by diffusion and increasing the efficiency and control of the catalytic processes in the multienzymatic aggregate [8]–[12]. Substrate channelling can occur within protein matrix channels or along the electrostatic surface of the enzymes belonging to macromolecular complexes [13], [14].

In addition, reversible interactions of some enzyme aggregates with structural proteins and membranes are of common occurrence in eukaryotic cells, leading to the emergence of metabolic microcompartments within the soluble phases of cells [15]–[20].

Prokaryotic cells also exhibit microcompartments, but in this case they have outer shells which are composed of thousands of protein subunits and are filled with enzymes belonging to specific metabolic pathways in the interiors [21], [22].

Besides apart from forming complex catalytic associations, enzymes can exhibit oscillatory catalytic patterns which allow the temporal self-organization of metabolic processes.

During the last four decades, extensive studies of dynamical biochemical behaviors in cellular conditions both in prokaryotic and eukaryotic cells have revealed the spontaneous emergence of molecular oscillations in most of the fundamental metabolic processes. For instance, biochemical oscillations have been reported to occur in: NAD(P)H concentration [23], biosynthesis of phospholipids [24], cyclic AMP concentration [25], ATP [26] and other adenine nucleotide levels [27], intracellular glutathione concentration [28], actin polymerization [29], ERK/MAPK metabolism [30], mRNA levels [31], intracellular free amino acid pools [32], cytokinins [33], cyclins [34], transcription of cyclins [35], gene expression [35]–[39], microtubule polymerization [40], membrane receptor activities [41], membrane potential [42], intracellular pH [43], respiratory metabolism [44], glycolysis [45], intracellular calcium concentration [46], metabolism of carbohydrates [47], beta-oxidation of fatty acids [48], metabolism of mRNA [49], tRNA [50], proteolysis [51], urea cycle [52], Krebs cycle [53], mitochondrial metabolic processes [54], nuclear translocation of the transcription factor [55], amino acid transports [56], peroxidase-oxidase reactions [57], protein kinase activities [58] and photosynthetic reactions [59].

The transition from simple periodic behavior to complex oscillatory phenomena, including bursting (oscillations with one large spike and series of secondary oscillations) [60] and chaos (irregular oscillations), is often observed in metabolic behaviors [61].

In the conditions prevailing inside the cell, the oscillations represent one of the most striking manifestations of dynamic behaviour not only of qualitative, but also, of quantitative importance in cell metabolic systems; e.g., considering only the transcription processes, it has been reported that at least 60% of all gene expression in Saccharomyces cerevisae oscillates with an approximate period of 300 min [62] and at least 10% of the rest of cellular transcripts oscillate in a circadian manner [63].

This new type of supramolecular self-organization that operates in far-from-equilibrium conditions was called dissipative structure by Prigogine [64], [65] and the enzymatic functional structures that provide the temporal self-organization of metabolism find their roots in the many regulatory processes that control the dynamics of the enzymes that belong to them [65].

Dissipative enzymatic complexes are advantageous thermodynamically biochemical structures, which acting as individual catalytic entities forming unique, well-defined dynamical systems and their activity are autonomous with respect to the other enzymatic associations [1]. Each set of dissipatively-structured enzymatic associations acts as a metabolic dynamic subsystem in which molecular oscillations and steady state patterns may emerge spontaneously. These enzymatic sets called metabolic subsystems form a reactive entity as a whole and seem to constitute the catalytic basic elements of the cellular metabolism [1].

Summing up, extensive studies have shown that (1) the functional enzymatic associations which operate in far-from-equilibrium conditions forming dissipative catalytic entities, (2) the substrate channeling and (3) the microcompartmentalization of the metabolic processes are the principal ways of structural organization of the eukaryotic cell metabolism [1]. These elements are the basis for more complex biomolecular self-organizations at superior structural and functional levels as for example the Intracellular Energetic Units (ICEU) [66] and the synaptosomes [19].

The first model of a Dissipative Metabolic Network was developed in 1999 [67]. Essentially, these networks are open systems formed by a set of metabolic subsystems which are interconnected by substrate fluxes and regulatory signals (allosteric and covalent modulations).

The catalytic activity of the allosteric enzymes is modulated through the noncovalent binding of a specific metabolite at a different location from the catalytic site, provoking alterations of the metabolic state in an interval of seconds. Such types of modulation may be both positive (activation of their catalytic rates) and negative (inhibitory modulators). The regulation by means of the covalent interactions can originate “all-or-nothing” types of answers [68].

In agreement with experimental observations [1], the emergent output activity of the enzymatic subsystems in the dissipative networks may be oscillatory or steady state and comprises an infinite number of distinct activity regimes.

Each dissipative network can be considered as a super-complex dynamic structure which integrates a set of different dynamic systems (the metabolic subsystems) forming a unique, well defined, deterministic, dynamical super-system.

The first numerical studies with dissipative metabolic networks allowed to observe a singular spontaneously self-organized Systemic Metabolic Structure, characterized by a set of different enzymatic associations always locked into active states (metabolic core) while the rest of metabolic subsystems presented on-off dynamics. When a metabolic subsystem is in an-off state for a long time it can be turned on under specific metabolic conditions. In these numerical works it was also suggested that the systemic metabolic structure could be an intrinsic characteristic of metabolism, common to all living cellular organisms [67], [69].

Afterward, 2004 and 2005, several studies carried out implementing flux balance analysis in experimental data produced new evidences of this global functional structure [70], [71], [72]. Specifically, it was observed a set of metabolic reactions belonging to different anabolic pathways which remain active under all investigated growth conditions. The rest of the reactions belonging to different pathways remain only intermittently active. These global catalytic processes were verified for *Escherichia coli*, *Helicobacter pylori*, and *Saccharomyces cerevisiae* [71], [72].

The metabolic core forms a single cluster of permanently connected metabolic processes where the activity is highly coordinated. Two types of reactions are present in the metabolic core: the first type is essential for biomass formation in both optimal and suboptimal growth, while the second type of reactions is required only to assure optimal metabolic performance [71], [72].

More recently, extensive analyses with different dissipative metabolic networks have shown that the fundamental factor for the spontaneous emergence of this global self-organized enzymatic structure is the number of enzymatic dissipative associations (metabolic subsystems) [73]. Moreover, it has been observed that the Systemic Metabolic Structure forms a unique dynamical system, in which self-organization, self-regulation and persistent properties may emerge [74].

In order to investigate the functional importance of the metabolic core we have studied different catalytic time series belonging to a particular dissipative metabolic network. The data have been analyzed using information-based dynamics tools, such as Pearson's correlation and Transfer Entropy (TE).

Pearson correlations allow for a straightforward quantification of statistically dependencies between pairs of metabolic subsystems.

TE allows for a quantification of how much the temporal evolution of the activity of one metabolic subsystem helps to improve the future prediction of another [75]–[79] and therefore, here, we have been able to analyze which metabolic subsystems influences which, and in this way, it is possible to evaluate the effective connectivity of the dissipative metabolic networks.

In this paper we have quantified essential aspects of the metabolic core functionality, and the results show that in the metabolic network, besides the classical topological structure characterized by the specific substrate fluxes, covalent modulation processes and allosteric signals a dynamical functional organization of effective connectivity emerges; it is characterized by significant variations of biomolecular information flows. Likewise, we have found that this organization of the effective information flows is modular and the dynamical changes between the catalytic modules correspond to metabolic switches which allow critical transitions in enzymatic activity.

The metabolic core, the modules of effective connectivity and the functional switches seem to be fundamental elements in the self-regulation of the Systemic Metabolic Structure.

## Materials and Methods

### 1. Dissipative Metabolic Networks

As said in the Introduction section, experimental observations have revealed that enzymes may form functional catalytic associations in which a new type of dissipative supramolecular self-organization may emerge [1], [64], [65].

We have called metabolic subsystems (MSb) or enzymatic subsystems to these groups of dissipatively structured enzymatic associations in which transitions between molecular oscillations and steady states may emerge spontaneously [1]. Each subsystem forms an enzymatic entity as a whole, in which the catalytic activity is autonomous with respect to the other functional enzymatic associations.

A Dissipative Metabolic Network (DMN) is an open system formed by a given set of enzymatic subsystems interconnected by substrate fluxes and regulatory signals, which may be of three types: activatory (positive allosteric modulation), inhibitory (negative allosteric modulation) and all-or-nothing type (which correspond with the regulatory enzymes of covalent modulation). Certain enzymatic sets may receive an external substrate flux.

The regulatory signals come from any subsystem of the network and do not require any flux relationship.

Each subsystem transforms the input substrate fluxes and regulatory signals into the output catalytic activity. The input-output conversion is performed in two stages. In the first one, the input fluxes are transformed in an internal enzymatic activity of the subsystem by means of flux integration functions. In the second stage, the received regulatory signals modify the internal enzymatic activity converting it into output catalytic activity.

The flux integration functions are based in the quantitative catalytic studies of the amplitude and frequency of the glycolytic patterns obtained by Goldbetter and Lefever in [80] under dissipative conditions. In the second stage, the internal enzymatic activity is modified by means of the regulatory signals integration, which depends on the combination of the received regulatory signals. Each regulatory signal has an associated regulatory coefficient which defines the intensity of its influence.

In agreement with experimental observations [1], the output activity of all the enzymatic subsystems may be oscillatory or steady state and comprise a very large number of distinct activity regimes. When a subsystem shows an activity with rhythmic behaviour the output catalytic activities present nonlinear oscillations with different levels of complexity, as it could be expected in cellular conditions. Therefore, in the subsystems a large number of transitions between periodic oscillations and steady-states including deterministic chaotic patterns may emerge. The mechanism that determines the complex catalytic behaviour is not prefixed by any part of the metabolic system. There are not rules that determine the network to present complex transitions in the output activities of the metabolic subsystems. The complex dynamic behaviours which spontaneously emerge in the metabolic network have their origin in the regulatory structure of the feedback loops, and in the nonlinearity of the constitutive equations of the biochemical system.

Numerous mathematical studies on metabolic rhythms have contributed to a better understanding of the functionality of the enzymatic subsystems in cellular conditions. Most of the functional biochemical studies have been carried out by means of systems of differential equations e.g., in the Krebs cycle [81], in the amino acid biosynthetic pathways [82], in the oxidative phosphorylation subsystem [83], in the glycolytic subsystem [84], in the transduction in G-protein enzyme cascade [85], in the gene expression [86], in the cell cycle [87]. Likewise, in order to understand the emerging dynamics in a single enzymatic set dissipatively structured we have also investigated the yeast glycolytic subsystem by means of a system of differential equations with delay [88]. In these studies we have analyzed different attractor dynamics linked to Hopf bifurcations [89], tangent bifurcations [90], the classical period-doubling cascade preceding chaos [91] and the multiplicity of coexisting attractors in the phase space [91], [92].

In all these studies, it is assumed that each metabolic subsystem forms a unique dynamical system governing by different attractors [1]. Therefore, the subsystems carry out their activity with autonomy between them and play distinctive and essential roles in the cell [65].

In a DMN, two kinds of attractors emerge: systemic and local [74].

The asymptotic behaviours of activities of all metabolic subsystems form systemic attractors in the phase space of the global metabolic system [74]. At the same time, the subsystems show local attractors which are defined by their local variables.

In a recent work, we have shown that the local attractors belonging to the enzymatic subsystems are not projections of the systemic attractors. The systemic attractors and the local attractors of the subsystems are topologically different [74].

Therefore, a dissipative metabolic network can be considered as a super-complex dynamic structure which integrates a set of different dynamic systems (the metabolic subsystems) forming a dynamical-super-system [74].

### 2. Subsystem activities

All the explicit details on how DMNs are constructed can be found in [68], [70], [73], [74] and are sketched in what follows.

Formally, we assume that the activity of the i-th enzymatic subsystem is defined by
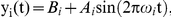
where 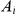 is the amplitude of oscillation, 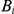 is the baseline and 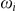 is the oscillation frequency. Moreover, to have 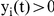 we assume that 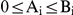 and the baselines and frequencies are bounded values, so there exists 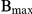 and 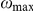 such that
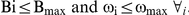
In this way, the activity of each subsystem 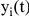 is characterized by three variables 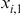, 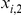 and 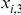, with values between 0 and 1 such that
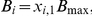


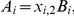


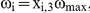
A subsystem is inactive when 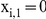, and is in a steady state when 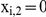 or 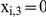.

We fix 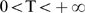 and define 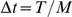 as the time interval during which the oscillations is maintained in the m-th time interval between 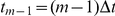 and 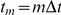. In that interval, the activity of the i-th subsystem is determined by the vector 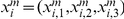 and the state matrix by
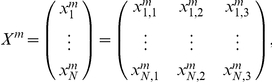
which characterizes the whole DMN system, with *N* the total number of subsystems.

### 3. Flux integration

Let us suppose that the i-th subsystem receives a flux from the j-th. Its internal activity represented by 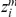 will be computed by three flux integration functions:
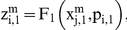


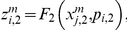


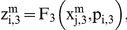
Where 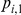, 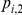 and 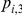 are parameters associated to the flux integration function which are characteristic of each metabolic subsystem, and the 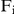 are piecewise linear approximations for nonlinear functions obtained by Goldbeter and Lefever in [80]. In this paper, the functions will be the following:
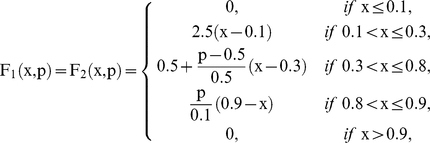
and
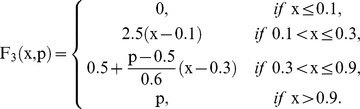
When a subsystem receives different fluxes from at least two subsystems, we compute the arithmetic mean of the F-values previously calculated.

### 4. Regulatory signal integration

In this second stage, the internal activity values are modified using the signal integration functions, which depend on the combination of the received regulatory signals and their corresponding parameters (regulatory coefficients). In the metabolic subsystems, the existence of some regulatory enzymes (both allosteric and covalent modulation) increases the interconnection among them. The allosteric enzymes present different sensitivities to the effectors, which can generate diverse changes on the kinetic parameters and in their molecular structure; likewise, the enzymatic activity of covalent modulation also presents different levels of regulation. These effects on the catalytic activities are represented in the dynamical system by the regulatory coefficients and consequently each signal has an associated coefficient which defines the intensity of its influence.

There exist three kinds of signal integration functions:

-Activation function AC.
-Inhibition function IN.
-Total inhibition function TI.

In this way, to compute 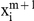 from 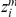 the i-th subsystem receives enzymatic regulatory signals from r subsystems and they work sequentially computing
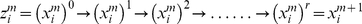
where each step depends on the signal type. From 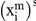 to 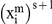 if the signal is AC and is received from the j-th MSb
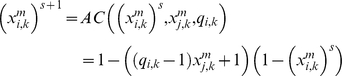
for *k* = 1, 2, 3 and 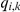 are regulatory coefficient to each allosteric activity signal which represents the sensitivity to the allosteric effectors.

If the allosteric signal is inhibitory
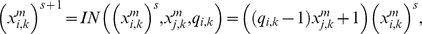
and, finally, if the signal is of the total inhibition type
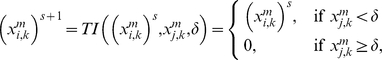
where δ, the threshold value, is the regulatory coefficient associated to each enzymatic activity signal of covalent modulation which defines the intensity of its influence.

### 5. Metabolic network generation

First, we have fixed the following elements as control parameters: (1) 18 subsystems in the DMN, (2) three substrate input fluxes for each subsystem (each MSb can receive a maximum of three substrate fluxes and it is not restricted the number of flows leaving of them), (3) three input regulatory signals for each metabolic subsystem and (4) the same number of signals per class (allosteric activation, allosteric inhibition and covalent modulation). Certain metabolic subsystems may receive a substrate flux from the exterior and we have arbitrarily fixed the MSb3 and the MSb10 for this function.

Having fixed these elements, the structure of the network has been randomly configured, including: (1) the topology of flux interconnections and regulatory signals, (2) the 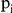 parameters associated to the flux integration functions, (3) the 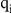 regulatory coefficients to the allosteric activities, and (4) the values of the initial conditions in the activities of all metabolic subsystems (Table S1).

The values of 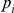 and 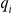 are random numbers between 0 and 1. The changes in the parameters 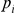 modify the flux integration functions. The values of 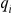 represents the influence level of the allosteric regulatory signals (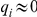 for a low level and 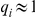 for a high level). The random values of the parameters 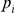 and 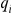 originate metabolic networks with a great variety of catalytic activities in each subsystem.

We have taken the constants 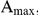 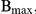 and 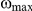 equal to 2, and δ, = 0.54 the threshold value of the regulatory coefficient associated with the covalent modulation signal which defines the intensity of its influence.

Finally, given *T* and *M* we calculate the activity matrices 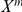 for *m* = 1,…, *M* using the flux integration functions and regulatory signals.

After numerical integration of the selected network, we generate a discrete time-series for the 3-tuples 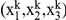. For all cases, the series of baseline, amplitude and frequency are analyzed after 1000 transitions.

### 6. Representation of the activity of the metabolic subsystems

We consider a number *N* of transitions. At the *k-th* iteration-step we suppose that the oscillation is harmonic, that is, the activity of the subsystem is described by a function of the form 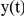 = 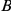
+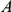
sin(2π
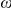
t), where 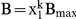, 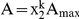 and 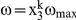, and where 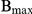 and 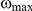 are fixed parameters independent on the stage number and on the subsystem. The duration of the harmonic oscillation is a given parameter 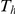 independent also on the stage and on the subsystem. Along the two stages, a mixed transition regime is maintained with a duration 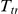 which is independent of the stage number and of the subsystem. If the transition goes from the *k-th* stage to the *(k+1)-th* stage then, during the 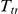 seconds of the transition regime, the activity is given by a function of the form 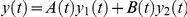, where 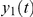 is the activity corresponding to the prolongation in time of the previous harmonic activity in the *k-th* stage, and 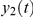 is the back-propagation in time of the subsequent harmonic activity in the *(k+1)-th* stage. The numbers 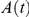 and 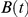 are time dependent and indicate the weights with which the activities of the subsystem in the previous and posterior stage are present during the transition time. At the beginning of the transition, say 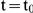, 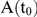 is 1 and 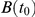 is 0, and at the end of the transition, say 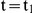, 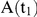 is 0 and 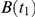 is 1. At the rest of the transition times 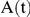 and 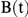 vary according to 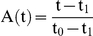, 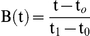. Finally, during the transition time the activity is given by
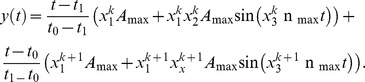
The transition regimes are combinations of two harmonic oscillations with nonconstant coefficients 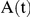 and 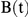 depending on time. Thus, the introduction of these transition regimes provokes the emergence of nonlinear oscillatory behaviors, both simple and complex.

### 7. Example of a simple DMN

We will consider a simple MN formed by two subsystems arranged in series with two feedback loops of regulatory signals. The MSb1 is activated by the second subsystem and the MSb2 is totally inhibited by the first subsystem when the MSb1 activity reaches a determinate threshold value. The input flux value of the MSb1 is 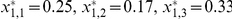, with 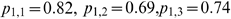. Parameter values for the integration functions of MSb2 are: 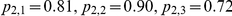. The catalytic dissipative element MSb1 is activated by the second MSb, with 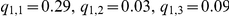 and the MSb2 is totally inhibited by MSb1, with a threshold δ = 0.18.

The initial state is
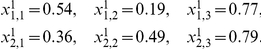
After the flux integration stage we reach an internal enzymatic activity with:
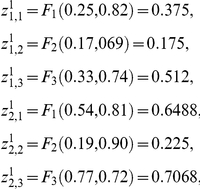
After the signal regulatory integration stage we obtain the following activity state
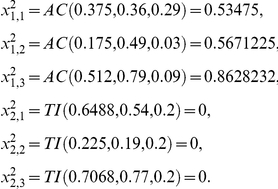
In the DMN the first metabolic subsystem will fall into a single active state, corresponding to a periodic oscillation, and the second subsystem is locked into an inactive state.

### 8. Pearson correlation

Pearson correlation is the simplest manner to quantify the statistical dependency between two dynamical variables. Values of Pearson correlation lie between −1 and 1. Two variables are perfectly correlated, meaning that when one variable increases the other does the same with the same proportion (one variable is up the other is up). The value −1 corresponds to the two variables being perfectly anti- correlated (one variable is up the other is down). The case zero-correlation corresponds to having two statistically independent variables.

The concept of synchronicity can also be related to correlations. High or low correlation values can be mapped to a high or low synchronicity between the two variables.

We have computed pairwise Pearson correlations between time series of amplitude of enzymatic activity. For each pair of time series, we shuffled the series to remove any statistical dependence between the two variables. After 50 repetitions of the shuffling experiment, the distribution of the correlation values is calculated. This distribution constitutes the null hypothesis since all dependencies have been removed by the shuffling procedure. Values of correlations with pvalue larger than 0.05 were reset to zero. In this way, we define the statistical significance of the correlations values given in Tables 1 and 2.

**Table 1:** Functional Connectivity based on Pearson Correlations: both stimuli S1 and S2.

	Sb1	Sb2	Sb3	Sb4	Sb5	Sb6	Sb7	Sb8	Sb9	Sb10	Sb11	Sb12	Sb13	Sb14	Sb15	Sb16	Sb17	Sb18
**Sb1**	1.00	0.34	−0.44	−0.01	−0.04	−0.09	−0.15	−0.30	−0.38	−0.44	0.26	−0.10	−0.19	0.65	0.00	−0.69	−0.52	−0.04
**Sb2**	0.34	1.00	−0.58	0.21	0.28	0.36	0.09	−0.04	−0.46	−0.60	0.34	0.11	0.03	0.12	0.00	0.00	−0.02	0.00
**Sb3**	−0.44	−0.58	1.00	−0.30	0.23	0.19	0.22	0.24	0.81	0.82	−0.55	0.40	0.19	−0.07	0.00	0.42	0.46	0.42
**Sb4**	−0.01	0.21	−0.30	1.00	−0.31	−0.33	−0.19	−0.02	−0.20	−0.37	−0.34	−0.67	−0.72	−0.19	0.00	−0.31	−0.48	0.14
**Sb5**	−0.04	0.28	0.23	−0.31	1.00	0.90	0.68	0.54	0.20	0.11	0.03	0.80	0.40	0.23	0.00	0.45	0.32	0.55
**Sb6**	−0.09	0.36	0.19	−0.33	0.90	1.00	0.48	0.27	0.33	0.19	0.12	0.80	0.56	0.25	0.00	0.43	0.46	0.53
**Sb7**	−0.15	0.09	0.22	−0.19	0.68	0.48	1.00	0.89	0.05	0.06	−0.22	0.59	0.20	−0.00	0.00	0.42	0.18	0.54
**Sb8**	−0.30	−0.04	0.24	−0.02	0.54	0.27	0.89	1.00	−0.07	−0.04	−0.43	0.38	−0.10	−0.27	0.00	0.45	0.00	0.36
**Sb9**	−0.38	−0.46	0.81	−0.20	0.20	0.33	0.05	−0.07	1.00	0.84	−0.29	0.35	0.37	0.09	0.00	0.28	0.48	0.48
**Sb10**	−0.44	−0.60	0.82	−0.37	0.11	0.19	0.06	−0.04	0.84	1.00	−0.26	0.35	0.48	0.11	0.00	0.39	0.59	0.34
**Sb11**	−0.10	0.11	0.40	−0.67	0.80	0.80	0.59	0.38	0.35	0.35	0.09	1.00	0.71	0.19	0.00	0.53	0.56	0.39
**Sb12**	−0.10	0.11	0.40	−0.67	0.80	0.80	0.59	0.38	0.35	0.35	0.09	1.00	0.71	0.19	0.00	0.53	0.56	0.39
**Sb13**	−0.19	0.03	0.19	−0.72	0.40	0.56	0.20	−0.10	0.37	0.48	0.53	0.71	1.00	0.21	0.00	0.50	0.74	0.06
**Sb14**	0.65	0.12	−0.07	−0.19	0.23	0.25	−0.00	−0.27	0.09	0.11	0.19	0.19	0.21	1.00	0.00	−0.36	−0.08	0.28
**Sb15**	0.00	0.00	0.00	0.00	0.00	0.00	0.00	0.00	0.00	0.00	0.00	0.00	0.00	0.00	0.00	0.00	0.00	0.00
**Sb16**	−0.69	0.00	0.42	−0.31	0.45	0.43	0.42	0.45	0.28	0.39	−0.03	0.53	0.50	−0.36	0.00	1.00	0.75	0.03
**Sb17**	−0.52	−0.02	0.46	−0.48	0.32	0.46	0.18	0.00	0.48	0.59	0.17	0.56	0.74	−0.08	0.00	0.75	1.00	0.14
**Sb18**	−0.04	0.00	0.42	0.14	0.55	0.53	0.54	0.36	0.48	0.34	−0.39	0.39	0.06	0.28	0.00	0.03	0.14	1.00

Density (%): 86.728, Mean: 0.200, Std. Dev.: 0.418.

**Table 2:** Functional Connectivity based on Pearson Correlations: only stimulus S1.

	Sb1	Sb2	Sb3	Sb4	Sb5	Sb6	Sb7	Sb8	Sb9	Sb10	Sb11	Sb12	Sb13	Sb14	Sb15	Sb16	Sb17	Sb18
**Sb1**	1.00	0.50	−0.14	0.18	0.11	0.05	−0.21	−0.25	−0.37	−0.23	−0.37	0.30	−0.39	0.73	−0.18	−0.56	0.04	0.06
**Sb2**	0.50	1.00	−0.16	0.11	0.12	0.11	0.30	0.05	−0.29	−0.43	0.23	0.29	0.09	0.31	−0.09	−0.02	0.30	0.09
**Sb3**	−0.14	−0.16	1.00	−0.31	0.14	0.01	0.11	0.12	0.55	0.24	−0.11	0.23	0.03	−0.20	0.46	0.00	−0.27	0.24
**Sb4**	0.18	0.11	−0.31	1.00	0.04	0.29	−0.15	0.00	−0.10	−0.51	−0.51	−0.11	−0.54	−0.00	−0.29	−0.40	−0.10	0.53
**Sb5**	0.11	0.12	0.14	0.04	1.00	0.81	0.80	0.81	−0.10	−0.32	0.11	0.84	−0.04	0.00	−0.10	0.28	0.42	0.21
**Sb6**	0.05	0.11	0.01	0.29	0.81	1.00	0.61	0.59	0.27	−0.20	0.04	0.66	0.03	0.02	0.23	0.15	0.42	0.60
**Sb7**	−0.21	0.30	0.11	−0.15	0.80	0.61	1.00	0.88	−0.09	−0.34	0.53	0.68	0.30	−0.26	−0.10	0.60	0.50	0.00
**Sb8**	−0.25	0.05	0.12	0.00	0.81	0.59	0.88	1.00	−0.18	−0.45	0.21	0.62	0.00	−0.40	−0.27	0.53	0.25	−0.04
**Sb9**	−0.37	−0.29	0.55	−0.10	−0.10	0.27	−0.09	−0.18	1.00	0.56	0.05	−0.06	0.34	−0.18	0.90	0.03	−0.07	0.63
**Sb10**	−0.23	−0.43	0.24	−0.51	−0.32	−0.20	−0.34	−0.45	0.56	1.00	0.17	−0.13	0.60	0.26	0.65	0.26	0.10	0.08
**Sb11**	−0.37	0.23	−0.11	−0.51	0.11	0.04	0.53	0.21	0.05	0.17	1.00	0.13	0.80	−0.21	0.16	0.59	0.62	−0.22
**Sb12**	0.30	0.29	0.23	−0.11	0.84	0.66	0.68	0.62	−0.06	−0.13	0.13	1.00	0.15	0.20	−0.00	0.31	0.55	0.20
**Sb13**	−0.39	0.09	0.03	−0.54	−0.04	0.03	0.30	0.00	0.34	0.60	0.80	0.15	1.00	−0.03	0.46	0.72	0.59	0.02
**Sb14**	0.73	0.31	−0.20	−0.00	0.00	0.02	−0.26	−0.40	−0.18	0.26	−0.21	0.20	−0.03	1.00	0.04	−0.23	0.22	0.11
**Sb15**	−0.18	−0.09	0.46	−0.29	−0.10	0.23	−0.10	−0.27	0.90	0.65	0.16	−0.00	0.46	0.04	1.00	0.06	0.04	0.53
**Sb16**	−0.56	−0.02	0.00	−0.40	0.28	0.15	0.60	0.53	0.03	0.26	0.59	0.31	0.72	−0.23	0.06	1.00	0.46	−0.14
**Sb17**	0.04	0.30	−0.27	−0.10	0.42	0.42	0.50	0.25	−0.07	0.10	0.62	0.55	0.59	0.22	0.04	0.46	1.00	0.19
**Sb18**	0.06	0.09	0.24	0.53	0.21	0.60	0.00	−0.04	0.63	0.08	−0.22	0.20	0.02	0.11	0.53	−0.14	0.19	1.00

Density (%): 95.679, Mean: 0.190, Std. Dev.: 0.390.

### 9. State probabilities, Uncertainty and Shannon Entropy

Let consider that the enzymatic activity of a given metabolic subsystem as represented by 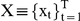, with 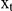 being the state at time t.

To compute probabilities, we considered the whole duration of the time series, and after counting the number of times in which the variable X is in state x, and dividing by time series length, we have the probability p(x), which is the normalized frequency of variable X in state x.

The uncertainty for a state x is defined as 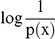; the smaller the probability for a given state x the less occurrence for that event, and therefore the more uncertain it is.

The Shannon Entropy 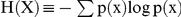 is the average uncertainty in the random variable X. The Entropy satisfies that 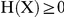 as 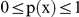. When the log is computed in base 2 (the case considered here) the Entropy is measured in bits.

The joint Entropy H(X,Y) between two random variables X and Y is just an extension of H(X) to 2-dimensions, i.e. 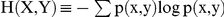, where the joint probability p(x,y) accounts for events in which the variable X is in the state x and simultaneously Y is in y. Analogously one can extend this Entropy to more than two variables.

### 10. Transfer Entropy

TE quantifies the information flow between pairs of metabolic subsystems. The oscillatory patterns of the biochemical metabolites might have information which can be read-out by the Transfer Entropy.

For a convenient derivation, let generally assume that the activity of a given pair of metabolic systems is represented by the two time series 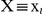 and 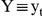, with 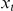 and 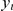 representing the instantaneous states of X and Y, so 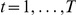. Let make the notation 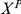 and 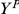 respectively for the pasts of X and Y and 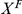 for the future of X.

Let now define 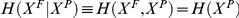 as the remaining uncertainty in 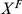 known 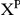, where the H-functions represent Shannon Entropies. 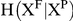 is a positive-definite quantity and has two extremes of interest; (1) The minimum corresponds to 
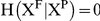
, when knowing 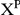 the uncertainty in 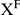 is completely determined and (2) the maximum, 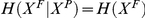, when knowing 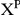 the uncertainty in 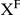 remains unchanged so both 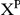 and 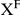 are statistically independent variables.

Analogously, 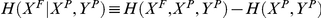 is the remaining uncertainty in 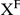 known 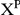 and 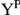. The difference 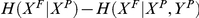 is by definition the transfer entropy from Y to X, which denoted by 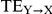 it is quantifying the amount (number of digits) of information that the inclusion of 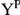 adds to the remaining uncertainty in 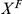 compared to the case in which solely is known 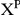. The term “information flow” comes from producing an uncertainty reduction when adding the second variable

Before obtaining an explicit formula for the TE, some comments about the definition of past and future events are required. First, the past of X might be related with more than a single time instant. This has been referred as the order of the Markov Process, and it refers to the number of past instants one should consider to compute the stationary probabilities for past events. Calling m the order, the future states in the time series will depend on the past m+1 events. Thus, we will take 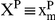 such as 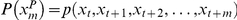 with 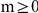 and analogously for 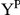. 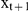 denotes the forward 1-lag time series and similarly 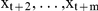 the forward 2-lags,…,m-lags. For the future time series we simply take 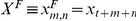 with 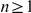.

With these two considerations, and using the definition of Shannon Entropy one obtains an explicit form for the Transfer Entropy
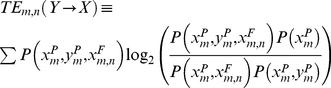
The results of the effective connectivity graphs shown here were computed for different values of m and n. In particular, we performed measures for m = 2,3,4,5 and n = 5,10 and the results did not change significantly.

It is important to remark that 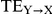 is different to 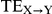, i.e., the effective connectivity is asymmetric, adding a directionality which accounts for a particular case of directed graphs, the graph of information flows between pairs of metabolic subsystems.

Alternatively to the Transfer Entropy, effective connectivity can be obtained using Granger Causality [93], which addresses how much the predictability of 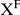 by looking only to 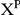 is improved when looking to both 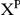 and 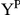. Recently, it has been proved that in the case of Gaussian variables both Transfer Entropy and Granger Causality are measuring exactly the same [94]. Therefore, the information flows based on Transfer Entropy and the Granger causality coincide for Gaussian variables.

### 11. Binning probabilities and statistical significance of TE

The bin size to compute probabilities was fixed to 4. We did not vary the bin size but addressed the statistical significance of our results. This was achieved by comparing the obtained values of 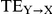 with the values obtained when considering a random permutation of the future of X, which named the shuffled-future of X constituted the null hypothesis. The non-zero values of TE in Tables are statistically significant; non-significant values were fixed to zero (pvalue = 0.05, Bonferroni correction for multiple comparisons with n = 50 shuffling experiments).

## Results

In order to investigate the functional importance of the metabolic core we have constructed a dissipative metabolic network of 18 subsystems, each of which represents a set of functionally associated and dissipatively structured enzymes, called indistinctly MSb, metabolic subsystem, enzymatic subsystem or subsystem (see Model and Methods section for more details).

**Figure 1:**
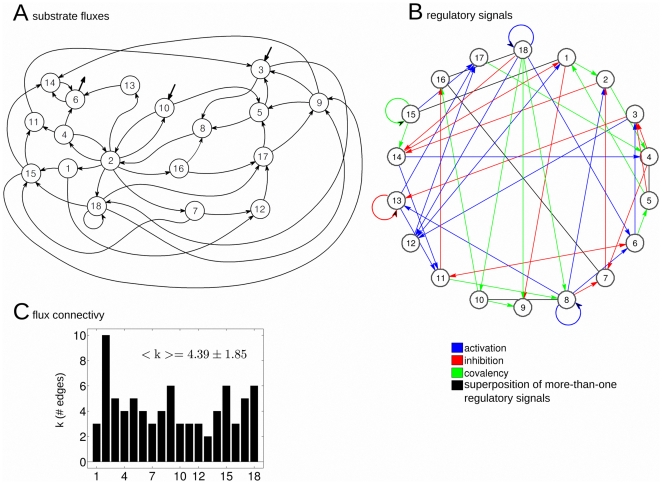
Topological structure of the dissipative metabolic network. The dissipative metabolic network formed by 18 catalytic subsystems in which the interconnection by substrate fluxes (figure A) and input regulatory signals (figure B) are depicted. Each subsystem represents a set of functionally associated enzymes which are dissipatively structured. Three classes of regulatory signals are considered: allosteric activation (blue), allosteric inhibition (red) and covalent modulation (green). Non-directed edges in black represent a superposition of more-than-one classes of signals. For instance, from MSb4 to MSb5 it exist a superposition of the three classes signals. The network might receive two substrate input fluxes S1 and S2, applied respectively to MSb3 and MSb10 with constant values of S1 = 0.54 and S2 = 0.16 (represented in the graph by two in-ward arrows). A (bottom): Black bars represent the total number of connections per subsystem (degree). Subsystem MSb2 is the hub, the node with a bigger number of edges.

Figure 1 illustrates the topological structure of the metabolic network which exhibits a complex organization of substrate fluxes and regulatory signals. The different quantitative characteristics of this dissipative metabolic network are given in Tables S1 and S2.

Three types of biochemical signals are considered in the network: activatory (positive allosteric modulation), inhibitory (negative allosteric modulation) and an all-or nothing type (corresponding to the regulatory enzymes of covalent modulation). Some enzymatic subsystems exhibit different regulatory feedback loops. Likewise, it can be observed that the MSb18 presents a self-catalytic process.

Regulatory signals come from any subsystem of the network and do not require any flux relationship.

The profile of flux connectivity has an average of 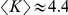 and exhibits some heterogeneity similar to the found in cellular conditions e.g., one subsystem has connectivity 10 while another has only K = 2).

Metabolic networks are open systems, and certain metabolic subsystems may receive a substrate flux from the exterior. Here, we have fixed the MSb3 and MSb10 for this function which receive the constant substrate inputs of S1 = 0.54 and S2 = 0.16.

### 1. Catalytic dynamic behaviours

First, we have studied the catalytic dynamical patterns that emerge under the two simultaneous external stimuli S1 and S2.

**Figure 2:**
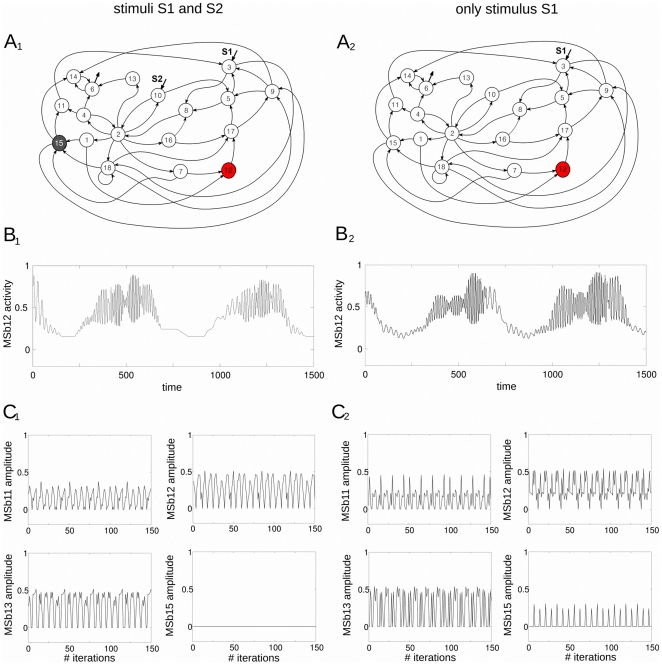
Dynamic catalytic patterns of the dissipative metabolic network. The network was perturbed by two different external conditions: (I) two simultaneous substrate input fluxes S1 and S2 (left column in panel) and (II) only with the stationary stimulus S1 (right column). (A_1_) Under the external condition I, a Systemic Metabolic Structure spontaneously emerges in the network in which the enzymatic subsystem MSb12 is always activity (metabolic core), the catalytic subsystem MSb15 is inactive, whereas the rest of enzymatic sets exhibit on-off changing states. (A_2_) In the condition II the network preserves the Systemic Metabolic Structure exhibiting flux plasticity which involve persistent changes in all the catalytic patterns (see B and C) and structural plasticity which results in a persistent change in the dynamic state of the subsystem MSb15 with a transition from an off to an on-off changing state. (B_1_) Example of the enzymatic activities of the enzymatic set MSb12 (metabolic core) with large number of different catalytic transitions between periodic oscillations and steady-states emerge in the network under condition I. (B_2_) In the condition II, persistent changes in the catalytic activity of the subsystem MSb12 can be observed (flux plasticity). (C_1–2_) During the metabolic self-regulation to the two external conditions changes for the amplitude of the enzymatic sets activities can be observed (flux plasticity).

Under this stimulation condition, a Systemic Metabolic Structure emerges spontaneously in the network in which the MSb12 is always in an active state (metabolic core), the MSb15 is always inactive whereas the rest of subsystems exhibit intermittently catalytic activities (on-off changing states) see figure 2A_1_.

The active subsystems present complex output catalytic patterns with large periodic transitions between oscillatory and steady state behaviours (105 transitions per period). Figure 2B_1_ shows a representative time series activities belonging to the MSb12 exhibiting 30 transitions between oscillatory and steady state behaviours.

Next, we have removed the external stimulus S2 and considered only the stationary input flux of substrate S1.

Under this new stimulation condition, the same network undergoes a drastic reorganization of its catalytic dynamics showing flux plasticity which involve persistent changes in all the catalytic activities (see figure 2B_1_–B_2_ and 2C_1_–C_2_), and structural plasticity which imply a persistent change in the state of the MSb15 (in the first conditions with the stimuli S1 and S2, the MSb15 was previously inactive and now is locked in an on-off changing dynamics), see figure 2A_2_–C_2_.

After the external perturbation with one stimulus S1, all subsystems present complex periodic activity with 350 different transitions between oscillatory and steady state behaviours (in figure 2B_2_ an example of 30 transitions in the catalytic activity of the MSb12 can be observed).

Despite the drastic catalytic changes observed in the time evolution of the dynamics of the subsystems, the network preserves its Systemic Metabolic Structure, i.e., the MSb12 is the metabolic core and the rest of subsystems continue exhibiting an intermittently active dynamics.

The complex dynamic behaviors which spontaneously emerge in the network have their origin in the regulatory structure of the feedback loops, and in the nonlinearity of the constitutive equations of the system. Therefore, the mechanism that determines these large transitions is not prefixed in any of the parts of the metabolic system and no rules determine the system to present complex transitions in the output activities of the metabolic subsystems.

Next, we have used the amplitude of the different catalytic patterns (see some examples in figure 2C) to study both functional connectivity based on Pearson correlation and effective connective based on Transfer Entropy (details below).

### 2. Functional connectivity based on Pearson correlation

Pearson correlation allow for a straightforward quantification of statistically dependencies between pairs of dynamical variables (further details in Model and Methods section).

The results of the Pearson correlation analysis are given in Tables 1 and 2 (pvalue<0.05, n = 50 experiments, Bonferroni correction).

**Figure 3:**
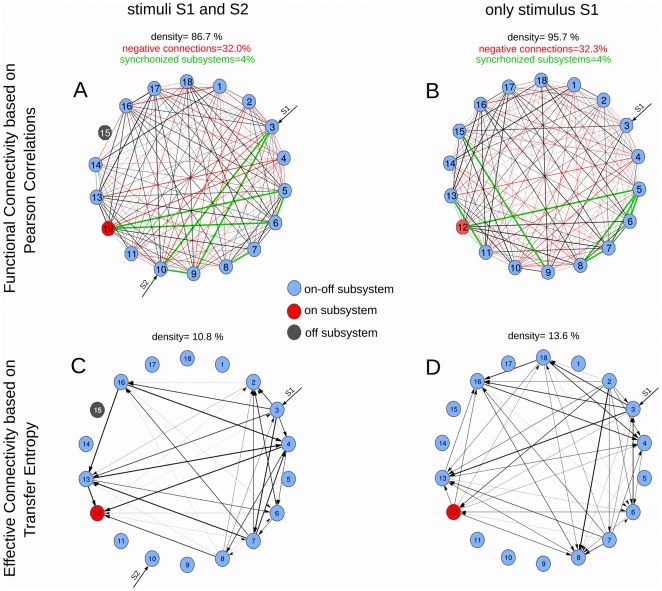
Functional correlations vs Effective connectivity. (Top): Functional connectivity based on Pearson correlations. A connection in the graph is corresponding to have a statistical significant correlation between two variables. Note that the correlation is a symmetric measure, so the edge is non-directed. The edge thickness for each graph is proportional to the correlation values given in Tables 1 and 2. About the 32% of the total connections are negative (depicted in red). In black we plotted positive correlations, in red negative correlations and in green the synchronized subsystems (see text for details). The high density of connections is an evidence of the systemic organization of the metabolic network. (Bottom): Effective connectivity based on Transfer Entropy. Because the TE is a non-symmetric measure now edges are directed. Similar to correlations, the edge thickness is proportional to the values of TE given in Tables 3 and 4. The TE analysis shows a dynamical functional organization of effective connectivity in the metabolic network. (Top and Bottom): Different enzymatic subsystems are plotted in different colors, light-blue for *on-off* enzymatic sets, red for the *on* enzymatic subsystem (metabolic core) and dark-gray for the *off* metabolic subsystem, occurring only for MSb15. The density of the graph is defined as the number of significant connections divided by the possible number of connections. Both values of correlations and TE are statistical significant (pvalue<0.05, Bonferroni correction, n = 50 experiments).

The graph of the functional correlations is highly dense for both stimulation conditions, cf. figure 3 top panel, densities of 86.7% (figure 3A) and 95.7% (figure 3B). This density is defined as the number of significant connections divided all the possible connections. Such robustness preserving a high density of connections is an evidence of the systemic organization of the metabolic network as the dynamics of the different subsystems are statistically dependent each other.

The statistical dependencies existing in high correlation (or anti-correlation) values between pairs of subsystems is corresponding to having similar (or opposite) functionality. The high density of connections based on correlations is indicating that there are common functionalities among most of the pairs of subsystems (about a 90% of the total). Contrarily, the subsystems with zero-correlation (about a 10%) do not interact directly with each other, but they can make it through common nearest neighbors.

In figure 3 top panel, we plotted in black the positive correlations (about a 70% of the total) indicating variables with similar function and in red variables with negative correlations, with opposite function (about a 30%).

For the situation of both stimuli S1and S2 the MSb15 is an off subsystem, therefore there are no correlations with any of the subsystems.

It is important to remark that in the case of having very high values of correlation it is possible to extract information about the synchronization in the dynamics of pairs of subsystems. High values of correlation indicate that the two variables do either increase or decrease at the same time (phase-locked), thus in a synchronized manner. In a similar way, very low values of correlation identify to anti-synchronized subsystems. We have not found such high values though (the lowest value was −0.72). In order to detect good synchronization values, we have fixed a threshold of correlation equals 0.8. The percentage of synchronized subsystems pairs is about the 4% of the total (7 subsystems pairs of a total 153) and that percentage kept constant for the two stimulation conditions. However, although the percentage of synchronized pairs was the same, the group of subsystems being synchronized did change. Thus, for instance, for the stimulation condition I (two external stimuli) the core MSb12 was synchronized with subsystems MSb5 and MSb6 (correlations values of 0.80) and for condition II (one external stimulus) the core increased the synchronization to MSb5 (from 0.80 to 0.84) but de-synchronized with MSb6 (from 0.80 to 0.66), Therefore, the patters of synchronization among different enzymatic sets also changed with the experimental condition.

### 3. Effective connectivity based on Transfer Entropy

Transfer Entropy quantifies the uncertainty reduction in the future activity of some metabolic subsystems when adding information of others. Unlike Pearson correlations, the effective connectivity based on TE can distinguish causal relations between the catalytic activities. TE is measured in information bits (see Model and Methods section).

For the first stimulation condition, in which the network receives two simultaneous stimuli S1 and S2, the graph of effective connections is shown in figure 3C. The arrows of the graph illustrate that the TE has directionality the arrows thickness is proportional to the values of TE given in Table 3.

**Table 3:** Effective Connective based on Transfer Entropy: both stimuli S1 and S2.

	To1	To2	To3	To4	To5	To6	To7	To8	To9	To10	To11	To12	To13	To14	To15	To16	To17	To18
**From1**	0.000	0.000	0.000	0.000	0.000	0.000	0.000	0.000	0.000	0.000	0.000	0.000	0.000	0.000	0.000	0.000	0.000	0.000
**From2**	0.000	0.000	0.000	0.000	0.000	0.000	0.000	0.000	0.000	0.000	0.000	0.000	0.023	0.000	0.000	0.009	0.000	0.000
**From3**	0.000	0.114	0.000	0.104	0.000	0.004	0.173	0.000	0.000	0.000	0.000	0.000	0.113	0.000	0.000	0.116	0.000	0.000
**From4**	0.000	0.005	0.000	0.000	0.000	0.071	0.000	0.107	0.000	0.000	0.000	0.176	0.000	0.000	0.000	0.000	0.000	0.000
**From5**	0.000	0.000	0.000	0.000	0.000	0.000	0.000	0.000	0.000	0.000	0.000	0.000	0.000	0.000	0.000	0.000	0.000	0.000
**From6**	0.000	0.122	0.000	0.000	0.000	0.000	0.072	0.000	0.000	0.000	0.000	0.017	0.000	0.000	0.000	0.000	0.000	0.000
**From7**	0.000	0.082	0.000	0.157	0.000	0.000	0.000	0.005	0.000	0.000	0.000	0.000	0.173	0.000	0.000	0.102	0.000	0.000
**From8**	0.000	0.038	0.042	0.000	0.000	0.004	0.000	0.000	0.000	0.000	0.000	0.116	0.004	0.000	0.000	0.004	0.000	0.000
**From9**	0.000	0.000	0.000	0.000	0.000	0.000	0.000	0.000	0.000	0.000	0.000	0.000	0.000	0.000	0.000	0.000	0.000	0.000
**From10**	0.000	0.000	0.000	0.000	0.000	0.000	0.000	0.000	0.000	0.000	0.000	0.000	0.000	0.000	0.000	0.000	0.000	0.000
**From11**	0.000	0.000	0.000	0.000	0.000	0.000	0.000	0.000	0.000	0.000	0.000	0.000	0.000	0.000	0.000	0.000	0.000	0.000
**From12**	0.000	0.005	0.002	0.000	0.000	0.000	0.010	0.000	0.000	0.000	0.000	0.000	0.000	0.000	0.000	0.000	0.000	0.000
**From13**	0.000	0.000	0.000	0.178	0.000	0.076	0.000	0.000	0.000	0.000	0.000	0.173	0.000	0.000	0.000	0.000	0.000	0.000
**From14**	0.000	0.000	0.000	0.000	0.000	0.000	0.000	0.000	0.000	0.000	0.000	0.000	0.000	0.000	0.000	0.000	0.000	0.000
**From15**	0.000	0.000	0.000	0.000	0.000	0.000	0.000	0.000	0.000	0.000	0.000	0.000	0.000	0.000	0.000	0.000	0.000	0.000
**From16**	0.000	0.000	0.000	0.176	0.000	0.010	0.000	0.000	0.000	0.000	0.000	0.000	0.179	0.000	0.000	0.000	0.000	0.000
**From17**	0.000	0.000	0.000	0.000	0.000	0.000	0.000	0.000	0.000	0.000	0.000	0.000	0.000	0.000	0.000	0.000	0.000	0.000
**From18**	0.000	0.000	0.000	0.000	0.000	0.000	0.000	0.000	0.000	0.000	0.000	0.000	0.000	0.000	0.000	0.000	0.000	0.000

Density (%): 10.802, Mean: 0.079, Std. Dev.: 0.065, Max.: 0.179.

The analysis shows that there are only 9 metabolic subsystems with statistical significant TE connections (figure 3C). Each of these 9 subsystems are effectively connected with other 8 subsystems by information flows (except the core MSb12 which is connected to 7 enzymatic sets).

When comparing the TE connectivity to the Pearson correlation the density of connections goes down very considerably, the density is equal to 86.7% for correlation versus a 10.8% for TE (Tables 1 and 3), thus the effective connectivity based on TE is about 8 times less dense than the functional connectivity based on Pearson correlation, what it can be easily observed comparing figures 3A and 3C.

The maximum value of TE equal to 0.179 information bits is corresponding to the connection from MSb16 to MSb13. The mean value of TE connections is 0.079 and the standard deviation is 0.065 (cf. Table 3).

For the second stimulation condition, in which the stimulus S2 was suppressed, the values of TE are given in Table 4 and its graph is plotted in figure 3D.

**Table 4:** Effective Connectivity based on Transfer Entropy: only stimulus S1.

	To1	To2	To3	To4	To5	To6	To7	To8	To9	To10	To11	To12	To13	To14	To15	To16	To17	To18
**From1**	0.000	0.000	0.000	0.000	0.000	0.000	0.000	0.000	0.000	0.000	0.000	0.000	0.000	0.000	0.000	0.000	0.000	0.000
**From2**	0.000	0.000	0.110	0.136	0.000	0.120	0.123	0.377	0.000	0.000	0.000	0.208	0.124	0.000	0.000	0.102	0.000	0.045
**From3**	0.000	0.000	0.000	0.000	0.000	0.000	0.000	0.037	0.000	0.000	0.000	0.059	0.334	0.000	0.000	0.262	0.000	0.272
**From4**	0.000	0.000	0.000	0.000	0.000	0.085	0.000	0.021	0.000	0.000	0.000	0.000	0.000	0.000	0.000	0.000	0.000	0.000
**From5**	0.000	0.000	0.000	0.000	0.000	0.000	0.000	0.000	0.000	0.000	0.000	0.000	0.000	0.000	0.000	0.000	0.000	0.000
**From6**	0.000	0.000	0.244	0.000	0.000	0.000	0.000	0.078	0.000	0.000	0.000	0.112	0.000	0.000	0.000	0.000	0.000	0.000
**From7**	0.000	0.000	0.092	0.125	0.000	0.002	0.000	0.212	0.000	0.000	0.000	0.019	0.190	0.000	0.000	0.149	0.000	0.083
**From8**	0.000	0.000	0.000	0.000	0.000	0.000	0.000	0.000	0.000	0.000	0.000	0.000	0.128	0.000	0.000	0.000	0.000	0.000
**From9**	0.000	0.000	0.000	0.000	0.000	0.000	0.000	0.000	0.000	0.000	0.000	0.000	0.000	0.000	0.000	0.000	0.000	0.000
**From10**	0.000	0.000	0.000	0.000	0.000	0.000	0.000	0.000	0.000	0.000	0.000	0.000	0.000	0.000	0.000	0.000	0.000	0.000
**From11**	0.000	0.000	0.000	0.000	0.000	0.000	0.000	0.000	0.000	0.000	0.000	0.000	0.000	0.000	0.000	0.000	0.000	0.000
**From12**	0.000	0.000	0.000	0.066	0.000	0.000	0.000	0.153	0.000	0.000	0.000	0.000	0.124	0.000	0.000	0.134	0.000	0.059
**From13**	0.000	0.000	0.000	0.128	0.000	0.031	0.000	0.000	0.000	0.000	0.000	0.000	0.000	0.000	0.000	0.139	0.000	0.000
**From14**	0.000	0.000	0.000	0.000	0.000	0.000	0.000	0.000	0.000	0.000	0.000	0.000	0.000	0.000	0.000	0.000	0.000	0.000
**From15**	0.000	0.000	0.000	0.000	0.000	0.000	0.000	0.000	0.000	0.000	0.000	0.000	0.000	0.000	0.000	0.000	0.000	0.000
**From16**	0.000	0.000	0.000	0.267	0.000	0.008	0.000	0.171	0.000	0.000	0.000	0.000	0.000	0.000	0.000	0.000	0.000	0.000
**From17**	0.000	0.000	0.000	0.000	0.000	0.000	0.000	0.000	0.000	0.000	0.000	0.000	0.000	0.000	0.000	0.000	0.000	0.000
**From18**	0.000	0.000	0.000	0.253	0.000	0.008	0.000	0.096	0.000	0.000	0.000	0.000	0.104	0.000	0.000	0.281	0.000	0.000

Density (%): 13.580, Mean: 0.133, Std. Dev.: 0.089, Max.: 0.377.

By a simple inspection of figure 3D (one stimulus) versus 3C (two stimuli) the structure of the effective information flows is now more complex: (1) 10 of the 18 enzymatic sets have effective connections, 8 of the 10 are connected to 9 others, except the subsystems MSb3 and MSb4 which are connected to 8, (2) the density of effective connections grows from 10.802 (two stimuli) to 13.580 (one stimulus), and (3) not only the density increased but the connections made stronger (mean values grow from 0.079 to 0.133 and the maximum value of effective connectivity is 0.377 versus 0.179 information bits, this maximum goes from MSb2 to MSb8, thus appearing at a completely different location under two external stimulus where the maximum was from MSb16 to MSb13.

Therefore, the TE analysis reveals that the structure of effective information flows is dynamical and it strongly varies depending on the two external conditions.

**Figure 4:**
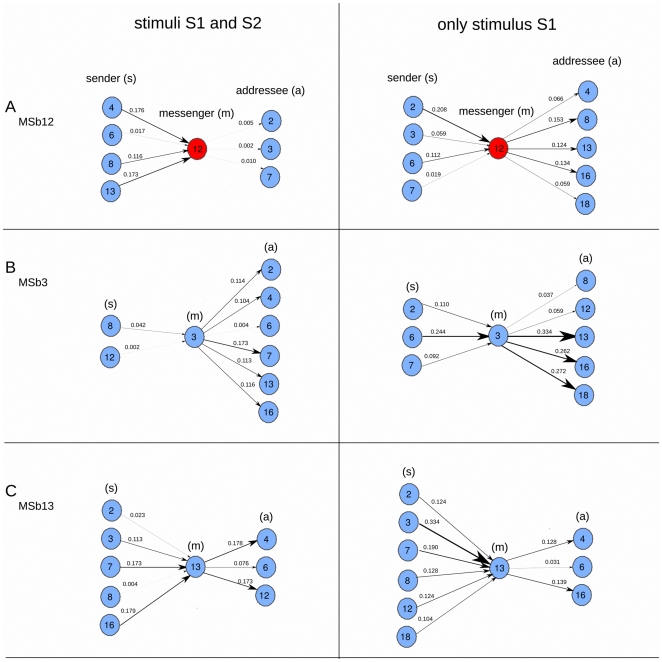
In-ward and Out-ward of bio-molecular information flows. Same information as bottom graphs in Fig. 3 but with another view point. For subsystems MSb12 (A), MSb3 (B) and MSb13 (C) we plotted the in-ward and out-ward TE for the two stimulation conditions (left and right columns). With the letters (s), (m) and (a) we are referring respectively to sender, messenger and addressee. The dynamical functional structure of effective information flows depends strongly on the two external conditions showing significant variations of bio-molecular information flows.

This is also further illustrated in the effective connectivity dynamics of the metabolic core (figure 4). One can see how (1) some connections preserved from switching the condition I (two stimuli) to II (one stimulus) e.g., from MSb6 to MSb12, but changed the TE values from 0.017 information bits to 0.112, (2) others preserved but inverted the directionality in time, e.g., the connection from MSb4 to MSb12 in condition I flipped its direction in condition II, and (3) some others were newly created e.g., the connection from MSb12 to MSb13 emerged in condition II. Thus, the paths in which the metabolic core is participating have drastic variations when changing the external conditions. Similarly, those changes depending on external conditions also occur for other enzymatic sets cf. figure 4B for MSb4 and figure 4C for MSb13.

**Figure 5:**
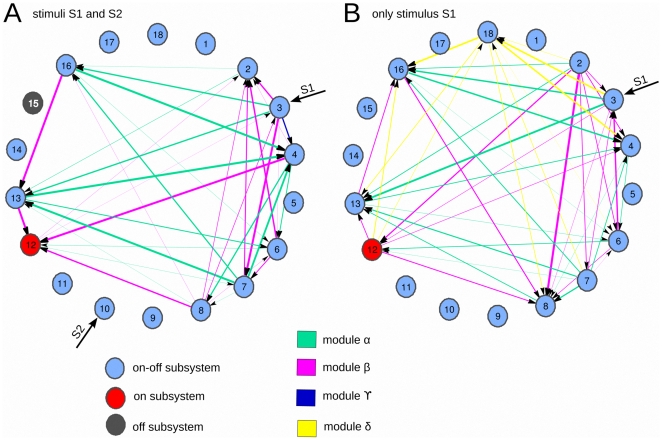
Modularity in the metabolic network. Different sets of enzymatic subsystems form modules of effective connectivity. A: both stimuli S1 and S2 are presented to the network. B: only stimulus S1 is presented. A,B: Same TE information as in Fig. 3 (bottom) but with a different viewpoint. We colored the edges grouping the enzymatic subsystems in different modules. In green we plotted the connections which were preserved for the two stimulation conditions (module alpha). In pink the connections existing in both A and B but with inverted directionality (module beta), thus flipping the causality direction given by the sign of the TE. In dark-blue we plotted the connection only existing in condition A, but disappearing in B (module gamma), and the opposite around, connections in B but not in A are plotted in yellow (module delta).

A further exploration shows a modular organization of the effective information flows, in which some sets of subsystems are clustered forming metabolic sub-networks. This is illustrated in figure 5. Thus, for the stimulation condition I (two stimuli), the network exhibits three different modules: 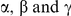 and the modular organization did not preserve but also changed with the external stimulation. In condition II (one stimulus) 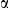 module preserved as also existed in condition I, the 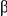 module preserved but inverted the time directionality and finally, a new δ module emerged, mainly connecting the Msb18.

The transitions between the modules provoke permanent changes in all the catalytic activities of subsystems (see figures 2B and 2C) and these metabolic switches are triggered by changes in the external conditions.

### 4. Total information flow

In order to extract new functionality based on TE, we have computed the total information flow, defined as the out-ward TE minus the in-ward TE per each enzymatic set. Thus, positive values are corresponding to sources and negative values to sinks of effective information (Tables 5 and 6).

**Table 5:** Total information flow: both stimuli S1 and S2.

MSb	1	2	3	4	5	6	7	8	9	10	11	12	13	14	15	16	17	18
**flow**	0.000	−0.333	0.580	−0.258	0.000	0.046	0.265	0.097	0.000	0.000	0.000	−0.466	−0.065	0.000	0.000	0.134	0.000	0.000

**Table 6:** Total information flow: only stimulus S1.

MSb	1	2	3	4	5	6	7	8	9	10	11	12	13	14	15	16	17	18
**flow**	0.000	−0.333	0.580	−0.258	0.000	0.046	0.265	0.097	0.000	0.000	0.000	−0.466	−0.065	0.000	0.000	0.134	0.000	0.000

One can observe that despite the drastic variations in connectivity structure reported in previous subsection, in terms of total information flow some metabolic subsystems did not change their attributes of being sources or sinks but preserved along the external stimulation condition.

**Figure 6:**
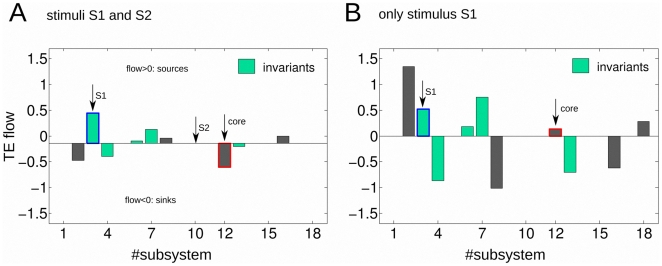
Transfer Entropy flow and metabolic functional invariants. A: both stimuli S1 and S2 are presented to the network. B: only stimulus S1 is presented. A,B: Transfer Entropy flow is defined as the out-ward TE minus the in-ward. Now connections are weighted, so the TE flow is different to the net TE in Fig. 5 where we only counted the number of connections in each direction with no consideration of the connections weight. Positive values of flow are corresponding with sources and negative values with targets or sinks. The subsystem attribute is the being of a source or a sink. Functional metabolic invariants are the subsystems with no changes on their attributes along the two stimulation conditions (left and right columns). This happened for enzymatic subsystems MSb3, MSb4, MSb6, MSb7 and MSb13 (bars colored in light green). The non-invariant subsystems were colored in gray. With a red line on subsystem MSb12 we marked the nucleus. With a blue line on subsystem MSb3, we marked that this subsystem in addition to be an invariant, it did neither change its magnitude, possibly encoding the external stimuli S1.

The subsystems which exhibit functional invariants are MSb3, MSb4 MSb6, MSb7 and MSb13. Three of them are sources (MSb3, MSb6 and MSb7), two of them sinks (MSb4 and MSb13) (Figure 6).

Likewise, there are 4 enzymatic sets that did change their attributes (Figure 6). Very remarkably, the metabolic core MSb12 is one of the non-invariants, inverting its function from being a sink in condition I (two external stimuli) to a source in condition II (one external stimulus).

Finally, the analysis shows that the invariant MSb3 did not change along the stimulation conditions not only its attribute of a source but either its magnitude, possibly encoding the stimulus S1.

## Discussion

In order to investigate the functional importance of the metabolic core, i.e. the enzymatic processes always in an active state, we have quantified essential aspects of the effective functional connectivity in a dissipative metabolic network of 18 metabolic subsystems, each one representing a set of enzymes functionally associated and dissipatively structured.

The metabolic network presents a topological organization of substrate fluxes with most of the nodes with low connectivity and a few hubs [95]. Likewise, the network includes an intricate structure of regulatory signals of allosteric and covalent modulations. Some enzymatic subsystems also exhibit regulatory feedback loops and one of them presents an autocatalytic process.

Under two stationary input fluxes of substrate which act simultaneously (external stimuli S1 and S2) the numerical analysis of the network shows that the enzymatic subsystems exhibit complex catalytic activities with large number of different transitions between oscillatory patterns and steady-states. Likewise, in the dissipative network spontaneously emerges a Systemic Metabolic Structure (SMS) in which the MSb12 is the metabolic core, the MSb15 is inactive, whereas the rest of subsystems exhibit intermittently active dynamics, i.e., on-off changing states.

Despite the complex activity observed in each subsystem with a large number of different catalytic transitions between periodic oscillations and steady-states, the network preserves the Systemic Metabolic Structure. These kinds of local catalytic dissipative responses (transitions between oscillatory patterns and steady-states) [1] and the global functional structure (one metabolic core, an MSb inactive and the rest of subsystems in an on-off changing state) are in accordance with experimental observations [71], [72], [96].

Once the dynamical catalytic behavior was studied, we next analyzed the Pearson correlation between pairs of time series of metabolic subsystems. The results showed that 1) the enzymatic sets are densely connected, 2) positive correlations are dominant versus negative correlations and 3) only a 4% of subsystems are synchronized. Consequently, it exists a systemic metabolic structure in which the different subsystems are statistically dependent each other. This systemic structure of correlations is highly dynamical, and it changes depending on external conditions.

But functional correlations do not imply effective connectivity, and therefore, Pearson correlations do not distinguish between causal and non-causal informational interactions [97].

For this reason, in the second analysis, we have used Transfer Entropy (TE) to establish the effective functional connectivity in the network. Concretely, we have applied this method for a quantification of how much the temporal evolution of the activity of one enzymatic subsystem helps to reduce the uncertainty in the future of another.

The analysis of TE shows that the network exhibits a singular structure of causal information flows in which about 50% of enzymatic sets included the metabolic core exhibit both effective connectivity flows and a high number of causal connections.

These levels of effective functional influence account for the contribution of each subsystem to the generation of the different catalytic behavior in other enzymatic sets and add directionality in the influence interactions between them.

The activity patterns of the biochemical activities have effective information which can be read-out by the TE. Therefore, in terms of the bio-molecular information processing, in the network about a 50% of subsystems have zero-TE but they are correlated and the rest of enzymatic sets have effective connectivity.

When the stimulation condition is changed the same network undergoes a drastic reorganization of its catalytic dynamics (1) exhibiting flux plasticity which involve persistent changes in all the catalytic patterns [72], [96], and (2) structural plasticity which result in a persistent change in the dynamic state of the MSb15 with a transition from an off to an on-off changing state [72], [96]. Under this new external stimulation, despite the activity transitions observed in the time evolution of the subsystems (see figure 2), the network preserves the systemic structure, i.e., the MSb12 is the metabolic core and the rest of subsystems continue in an on-off changing state.

It is interesting to remark that the network have adjusted the internal metabolic activities to the new external environmental change (the input flux of substrate S2 was removed in the conditions II) by means of flux plasticity and structural plasticity which has been experimentally observed in the metabolism of several organisms as the main systemic molecular mechanisms of adaptation to external perturbations [72], [94].

Depending on the stimulation conditions the network exhibits different both qualitative and quantitative patterns of effective information flows. From conditions I, two stimuli, to II, one stimulus, (1) the density of causal connections become greater (from 10.802 to 13.580), (2) the average of TE value increases notably (0.133 to 0.79) and (3) about 50% of enzymatic subsystems undergoes remarkable variations of the causal connections between them, i.e., some connections disappear, others new emerge, and those that preserves modify their TE values.

The metabolic cores also manifest qualitative and quantitative changes in the information flows, specifically, increasing the TE values and modifying the connectivity with several subsystems. For example, under the first perturbation (external stimuli S1 and S2) the core receives from MSb8 a causal information flow with a value of TE = 0.116 and when the input flux of substrate S2 is removed, the directionality of the signal reverses and the core sends to the MSb8 a information flow of a TE = 0.153 information bits.

The level of effective influence flows between the enzymatic subsystems is not always the same but varies depending on the external environment conditions. The dissipative metabolic network is self-regulated exhibiting for this an informative systemic organization, able to modify the catalytic kinetics of the all enzymatic sets.

Summing up, in addition to the network topological structure, characterized by the specific location of enzymatic subsystems, molecular substrate fluxes and regulatory signals, there is a functional structure of effective information flows which is dynamic and exhibits notable variations of the causal interactions.

In the third analysis, we have measured the total information flows. Positive values mean that the subsystems are sources of causality flows and negative flows are interpreted as sinks or targets. The results show that 5 subsystems do not change their attributes of effective functionality. Specifically, three enzymatic sets are sources, and two are sinks.

These five enzymatic subsystems present complex dynamic behaviours in their activities with large number of different transitions between periodic oscillations and steady-states (in total more than of 100 transitions per MSb was studied), however the obtained functional attributes, source or sink, are preserved during the catalytic activities generated under the two different external conditions and therefore these functional attributes seem to be invariants.

It is remarkable that the MSb3 did neither change the sign of the information flow nor its magnitude, which does not happen with any other one. From the biochemical point of view this enzymatic set is functionally important because it receives the external stimulus S1, and it regulates the metabolic core by means of an allosteric activation signal. Therefore, the invariant MSb3 directly transmits information to the core on the biochemical perturbations originated from the external environment. Possibly, the MSb3 encodes the stimuli S1, but this interesting issue it might require other additional studies.

These invariant constraints depend neither on the external environment conditions nor on the subsystem activity state, but only on the dynamic characteristics of the Systemic Metabolic Structure.

The presence of invariant properties in some subsystems indicates the profound functional constraints emerging in the catalytic processes which are operating under systemic conditions. The internal metabolic environment exhibits functional restrictions which seem to be attributable to the activity of the biochemical system as a whole. In an intuitive way, this idea was already announced by Claude Bernard, who can be considered one of the first systems biologists in history [98], [99].

In contrast with the five subsystems that develop functional invariants, the metabolic core and three more subsystems exhibit a flexible functionality able to be either a source or a sink of causal information.

Consequently, from the point of view of the information theory, three kinds of enzymatic associations emerge in the network, i.e., subsystems with zero-TE but correlated, invariant subsystems and un-constrained enzymatic subsystems.

The metabolic processes under systemic conditions form a global dynamic structure, highly interconnected, able to transmit information between its parts, in such a way that the activity of each enzymatic subsystem could be considered as an informative operation.

Each catalytic element of the network, in its subordination to substrate fluxes and regulatory signals generated by other metabolic subsystems, would perform three functions at the same time: signal reception, signal integration and acting as a source of new biomolecular information.

At global level, the transmission of information between the enzymatic subsystems provokes the emergence of a dynamical functional organization characterized by changing effective connectivity flows and functional attributes on the enzymatic subsystems (unconstrained enzymatic sets, invariant subsystems and subsystems with zero-TE but correlated). Therefore, the Systemic Metabolic Structure generates a complex network of functional constraints for the activities of the enzymatic sets establishing an informational hierarchy between them.

The emergent Systemic Metabolic Structure in the biochemical system is characterized by an effective functional organization which generates information flows between the metabolic subsystems, forcing them to be interlocked between themselves; i.e., each subsystem is conditioned to cooperate with others and have precise and specific activity regimes in concordance to the activity system as a whole. As a result of the overall process, the network operates as a complex information processing system which defines in every moment sets of biochemical instructions that makes each enzymatic set to evolve with a particular and precise catalytic pattern.

Lastly, our analyses also show that the organization of the effective information flows is modular. A detailed study of the TE allows inferring a different modular organization: (1) a set of effective connections between determined subsystems is preserved during both external conditions (Module 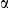), (2) a second sub-network of effective information flows exhibit reverse directionality (Module 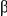) and the third set of connections emerge only in one of the considered external perturbations (Modules 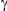 and 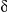) (see figure 5).

During the first perturbation of the network (simultaneous external stimuli S1 and S2) the diversity of the observed catalytic behaviors are systemically self-regulated by means of the Modules 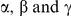. However, when it is only considered a stationary input flux of substrate S1, the network undergoes a dramatic reorganization of all catalytic dynamics exhibiting flux plasticity and structural plasticity; these drastic change are self-regulated by means of the Module 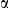, an emergent Module 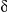 and the Module 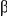, but now in this module all the directions of effective connectivity are reversing.

The modules seem to work as functional dynamic entities which allow to communicate very concrete enzymatic subsystems with well-defined others by means of effective information flows permitting high coordination and efficient catalytic regulations.

Under systemic conditions, our results show that the catalytic activities form functional modules which are highly self-regulated by means of biomolecular information flows in a way that different kinds of functional sub-networks coexist together. The change from a catalytic activity state to another persistent state is triggered by transitions at specific catalytic modules replacing of some functional sub-networks by another. These dynamic of change corresponds to metabolic switches which allow for critical transitions in the enzymatic activities.

The modular configuration of the enzymatic subsystems and the functional switches seem to be key elements to understanding the dramatic changes observed in the catalytic network when the external stimulus S2 is removed.

The network seems to operate as a complex information processing system which defines in every moment sets of biochemical instructions that makes to evolve with a particular and precise dynamical change of catalytic patterns. As a consequence of these systemic biomolecular processes the metabolic activities act as a whole able to self-regulate against external perturbations by means of effective functional flows and switches allowing flux plasticity and structural plasticity.

Metabolic self-regulation can be understood as a global process in which the enzymatic subsystems tend to reach a particular dynamics in their catalytic states, with autonomy against external factors, allowing for the integration of external stimuli and its adaptation of the metabolic system as a whole. This process is an emergent property originated by the complex dynamics of the global interactions and implies the modulation of each subsystem activity, driving the whole enzymatic behaviours over time and across changing circumstances. Contrarily to self-organization, the self-regulation does not seem to be a spontaneous process since it depends on local properties of subsystems and requires the processing of the information relative to the different states of all catalytic elements of the network.

Our results show that, at functional level, the metabolic switches are discrete transitions between enzymatic functional modules regulated by a systemic dynamics of the effective information flows.

At molecular level, a switch is a biochemical process in which determined enzymes can undergo a persistent change in their catalytic activity states. In experimental conditions it has been observed that, the molecular mechanisms implied in the switches generally depend on some kind of enzymatic activities [100] such as phosphorylations [101], acetylations [102]and methylations [103].

Molecular switches have been implicated in many types of metabolic processes including the transcriptional regulation [104], Warburg effect [105], [106], cell cycle [107], epigenetic processes [108], central carbon metabolism [109], DNA repair [110], growth cell metabolism [111] and T-Cell activation and apoptosis [112].

In this paper, we have quantified essential aspects of the Systemic Metabolic Structure and of the metabolic core functionality. In terms of the bio-molecular information processing, the enzymatic core is a hub which exhibits a high degree of connectivity with other subsystems, forming modules, and a wide range of effective information values depending on the external stimulation condition.

In addition, in the network we have observed an informational hierarchy (subsystems with zero-TE but correlated, functional invariant enzymatic sets and unconstrained metabolic subsystems) in which the metabolic core is an unconstrained subsystem in terms of information flow. In contrast with other subsystems the metabolic core does not present functional invariance but exhibits a flexible dynamic behavior acting as a source or a sink of causal information.

There are three other subsystems in the same category as the metabolic core (the MSb2, MSb8 and MSb16) which might have high relevance in the bio-molecular information processing. But this result would need further analysis.

In final summary, our analysis reveals that in addition to the biomolecular topological organization characterized by the specific location of enzymatic sets, substrate fluxes and regulatory signals, a global functional structure conformed by effective information flows emerge in the network which is dynamical and exhibits notable variations of the causal interactions.

Under systemic conditions, the dissipative catalytic processes form a functional self-organization of biomolecular information flows, becoming the enzymatic network in a complex information processing system.

The Systemic Metabolic Structure is not only characterized by a metabolic core, enzymes in an on-off changing state and a determinate biomolecular topological organization, but also forms a sophisticated structure of effective information flows which provides an informational hierarchy on the dissipative catalytic sets: subsystems with zero-TE but correlated, functional invariant enzymatic sets and unconstrained metabolic subsystems.

The functional structure of biomolecular information flows is modular and the dynamical changes between the modules correspond to metabolic switches which allow for critical transitions in the enzymatic activities.

These modules of effective connectivity and the functional switches seem to be important elements of the Systemic Metabolic Structure.

In the post-genomic era, the understanding of the elemental principles and quantitative laws that govern the self-organization and self-regulation of the catalytic processes under systemic conditions will be crucial to elucidate the functional architecture of the cell and the fundamental dynamics of cellular life.

## Supporting Information

Table S1 **Parameters of the dissipative metabolic network.** MSb: the number of metabolic subsystems; Reg. Signals: the topology of regulatory signals (+, allosteric activation; −, allosteric inhibition; −T, covalent modulation of total inhibition); Reg. Sign. Coef.: Coefficient values of the regulatory signals (belonging to the first, second and third regulatory signal); Fluxes in: the topology of flux interconnections; Flux Parameter: integration function parameters belonging to the first, second and third fluxes of the subsystems.
(DOC)

Table S2 **Initial conditions of the 18 metabolic subsystems.**
(DOC)
