# Supplementary material for: The Metabolic Core and Catalytic Switches Are Fundamental Elements in the Self-Regulation of the Systemic Metabolic Structure of Cells
Source: PLoS One. 2011 Nov 18;6(11):e27224. doi: 10.1371/journal.pone.0027224 (PMC3220688; doi:10.1371/journal.pone.0027224)
Supplement: Table S1 — Parameters of the dissipative metabolic network. MSb: the number of metabolic subsystems; Reg. Signals: the topology of regulatory signals (+, allosteric activation; −, allosteric inhibition; −T, covalent modulation of total inhibition); Reg. Sign. Coef.: Coefficient values of the regulatory signals (belonging to the first, second and third regulatory signal); Fluxes in: the topology of flux interconnections; Flux Parameter: integration function parameters belonging to the first, second and third fluxes of the subsystems. (DOC) [file pone.0027224.s001.doc]

**Table S1**

**Parameters of the dissipative metabolic network**

MSb: the number of metabolic subsystems; Reg. Signals: the topology of regulatory signals (+, allosteric activation; -, allosteric inhibition; -T, covalent modulation of total inhibition); Reg. Sign. Coef.: Coefficient values of the regulatory signals (belonging to the first, second and third regulatory signal); Fluxes in: the topology of flux interconnections; Flux Parameter: integration function parameters belonging to the first, second and third fluxes of the subsystems.

**TABLE S1**

| **MSb** | **Reg. Signals** | | | | | **Reg. Sign. Coef.** | | | | **Reg. Sign. Coef.** | | | | **Reg. Sign.Coef.** | | | |
| --- | --- | --- | --- | --- | --- | --- | --- | --- | --- | --- | --- | --- | --- | --- | --- | --- | --- |
| 1 | 5 T | 14 - | | 12+ | | .01 | .05 | .31 | | .32 | .82 | .31 | | .77 | .29 | .18 | |
| 2 | 7 - | 8 + | | 1 T | | .17 | .13 | .55 | | .52 | .65 | .91 | | .18 | .97 | .48 | |
| 3 | 4 - | 6 - | | 5 - | | .28 | .01 | .72 | | .97 | .34 | .37 | | .47 | .57 | .76 | |
| 4 | 14 + | 17 T | | 2 T | | .65 | .24 | .16 | | .19 | .79 | .88 | | .08 | .38 | .17 | |
| 5 | 4 T | 4 + | | 6 T | | .22 | .21 | .28 | | .65 | .61 | .24 | | .17 | .61 | .80 | |
| 6 | 8 + | 18 + | | 11 - | | .34 | .09 | .05 | | .42 | .82 | .70 | | .32 | .05 | .76 | |
| 7 | 16 T | 4 - | | 8 - | | .59 | .62 | .15 | | .56 | .01 | .12 | | .11 | .33 | .05 | |
| 8 | 18 T | 8 + | | 11T | | .32 | .14 | .14 | | .20 | .13 | .01 | | .20 | .37 | .16 | |
| 9 | 1 - | 18 T | | 10T | | .12 | .86 | .87 | | .16 | .04 | .05 | | .32 | .19 | .47 | |
| 10 | 8 T | 18 T | | 8 - | | .64 | .06 | .25 | | .03 | .07 | .38 | | .28 | .78 | .25 | |
| 11 | 14 + | 6 - | | 13+ | | .60 | .15 | .80 | | .08 | .31 | .19 | | .44 | .81 | .93 | |
| 12 | 1 + | 3 + | | 18+ | | .74 | .07 | .38 | | .28 | .17 | .10 | | .32 | .28 | .26 | |
| 13 | 13 - | 8 + | | 3 - | | .01 | .21 | .64 | | .44 | .31 | .97 | | .36 | .75 | .76 | |
| 14 | 15 T | 18 - | | 2 - | | .03 | .30 | .33 | | .43 | .24 | .69 | | .14 | .58 | .54 | |
| 15 | 1 - | 1 T | | 15T | | .19 | .98 | .29 | | .18 | .25 | .80 | | .50 | .16 | .11 | |
| 16 | 11 - | 10 T | | 7 + | | .96 | .24 | .97 | | .26 | .76 | .74 | | .10 | .29 | .01 | |
| 17 | 13 + | 15 + | | 12+ | | .33 | .63 | .47 | | .72 | .88 | .87 | | .74 | .65 | .43 | |
| 18 | 16 - | 18 + | | 16T | | .99 | .46 | .14 | | .99 | .51 | .58 | | .22 | .79 | .12 | |
| **MSb** | **Fluxes in** | | | | | **Flux Parameter 1º** | | | **Flux Parameter 2º** | | | | | **Flux Parameter 3º** | | | |
| 1 | 2 | | 2 | | 2 | .13 | .23 | .73 | .10 | | .35 | | .18 | .01 | .9 | | .26 |
| 2 | 10 | | 4 | | 8 | .29 | .56 | .48 | .77 | | .99 | | .63 | .56 | .47 | | .34 |
| 3 | 11 | | 9 | | 5 | .68 | .12 | .53 | .63 | | .78 | | .32 | .87 | .12 | | .68 |
| 4 | 2 | | 2 | | 2 | .94 | .08 | .17 | .47 | | .62 | | .99 | .17 | .66 | | .52 |
| 5 | 10 | | 9 | | 17 | .03 | .74 | .69 | .73 | | .81 | | .46 | .34 | .70 | | .47 |
| 6 | 14 | | 4 | | 13 | .34 | .31 | .55 | .58 | | .57 | | .88 | .68 | .22 | | .01 |
| 7 | 2 | | 2 | | 2 | .24 | .55 | .90 | .02 | | .19 | | .04 | .08 | .58 | | .11 |
| 8 | 16 | | 3 | | 5 | .69 | .61 | .61 | .75 | | .81 | | .88 | .47 | .09 | | .38 |
| 9 | 18 | | 15 | | 17 | .15 | .80 | .74 | .56 | | .93 | | .75 | .31 | .02 | | .31 |
| 10 | 2 | | 2 | | 2 | .82 | .68 | .24 | .19 | | .08 | | .55 | .89 | .38 | | .8 |
| 11 | 15 | | 15 | | 4 | .30 | .18 | .11 | .86 | | .97 | | .15 | .38 | .15 | | .97 |
| 12 | 1 | | 7 | | 7 | .25 | .74 | .52 | .94 | | .99 | | .58 | .81 | .31 | | .78 |
| 13 | 2 | | 2 | | 2 | .18 | .03 | .49 | .60 | | .78 | | .86 | .39 | .36 | | .35 |
| 14 | 9 | | 6 | | 15 | .87 | .64 | .80 | .62 | | .85 | | .37 | .93 | .6 | | .58 |
| 15 | 18 | | 1 | | 7 | .85 | .53 | .68 | .93 | | .74 | | .77 | .60 | .47 | | .71 |
| 16 | 2 | | 2 | | 2 | .58 | .27 | .72 | .27 | | .23 | | .86 | .06 | .13 | | .01 |
| 17 | 18 | | 16 | | 12 | .35 | .30 | .91 | .85 | | .67 | | .34 | .56 | .69 | | .93 |
| 18 | 3 | | 18 | | 2 | .98 | .58 | .58 | .52 | | .87 | | .56 | .87 | .43 | | .20 |
