# Supplementary material for: The Metabolic Core and Catalytic Switches Are Fundamental Elements in the Self-Regulation of the Systemic Metabolic Structure of Cells
Source: PLoS One. 2011 Nov 18;6(11):e27224. doi: 10.1371/journal.pone.0027224 (PMC3220688; doi:10.1371/journal.pone.0027224)
Supplement: Table S2 — Initial conditions of the 18 metabolic subsystems. (DOC) [file pone.0027224.s002.doc]

**Table S2**

**Initial conditions of the 18 metabolic subsystems**

**TABLE S2**

| **MSb** | **Initial Conditions** | | |
| --- | --- | --- | --- |
| 1 | .19 | .02 | .59 |
| 2 | .04 | .02 | .44 |
| 3 | .60 | .35 | .68 |
| 4 | .98 | .81 | .28 |
| 5 | .01 | .29 | .01 |
| 6 | .42 | .69 | .99 |
| 7 | .51 | .01 | .49 |
| 8 | .08 | .76 | .13 |
| 9 | .65 | .33 | .36 |
| 10 | .13 | .13 | .94 |
| 11 | .87 | .03 | .08 |
| 12 | .45 | .26 | .09 |
| 13 | .71 | .12 | .25 |
| 14 | .09 | .67 | .03 |
| 15 | .69 | .61 | .11 |
| 16 | .98 | .25 | .87 |
| 17 | .83 | .61 | .40 |
| 18 | .99 | .61 | .10 |
